# A teenager with CHD and coronavirus disease 2019

**DOI:** 10.1017/S1047951120002127

**Published:** 2020-07-06

**Authors:** Jakob Olfe, Maria Grafmann, Rainer Kozlik-Feldmann

**Affiliations:** Kinderkardiologie/Herzchirurgie für angeborene Herzfehler, Universitäres Herz- und Gefäßzentrum UKE Hamburg, Universitätsklinikum Hamburg Eppendorf, Hamburg, Germany

**Keywords:** Coronavirus disease 2019, CHD, severe acute respiratory syndrome coronavirus 2, mitral valve stenosis

## Abstract

A 16-year-old girl with history of treated congenital mitral valve disease and signs of respiratory infection was admitted to our paediatric cardiology department. She was tested positive for severe acute respiratory syndrome coronavirus 2. Despite her severe pre-existing cardiac conditions with pulmonary hypertension, atrial arrhythmias and mitral valve stenosis, the infection did not lead to any cardiac or pulmonary deterioration. In adults, cardiac co-morbidities are known risk factors for a severe course of coronavirus disease 2019 infections. This case illustrates that in children even severe cardiac disease is not necessarily associated with a severe course of coronavirus disease 2019.

A 16-year-old girl with history of treated congenital mitral valve disease and signs of respiratory infection was admitted to our paediatric cardiology department. She was tested positive for severe acute respiratory syndrome coronavirus 2.

She was born with a CHD with combined mitral valve disease (severe mitral valve insufficiency and mild mitral valve stenosis), resulting in giant left atrium with consecutive atrial flutter and fibrillation. At age 13, she had undergone a biological mitral valve replacement (Edwards, Perimount, 25 mm), a left atrial reduction plasty, and CryoMAZE procedure.

Over the last few months, she was complaining of increasing fatigue, chest pain, and pre-syncope on exertion. At her most recent cardiac evaluation 2 days before admission, transthoracic echocardiography showed severe stenosis and insufficiency of the mitral valve prosthesis. At baseline, the mean gradient over the valve was 13 mmHg and increased to 35 mmHg during exercise. Tricuspid regurgitation increased from mild to severe on exercise and revealed severe post-capillary pulmonary hypertension with a systolic right ventricular/pulmonary artery pressure of 80 mmHg (systemic non-invasive blood pressure 112/70 mmHg). Biventricular function was normal but exercise induced left ventricular T-wave inversion without wall motion abnormalities. The left atrium was hugely dilated (32 cm^2^ in four-chamber view). Twenty-four-hour Holter electrocardiogram showed atrial arrhythmia with junctional atrial beats and frequent monomorphic ventricular extrasystoles. No atrial flutter or fibrillation was recorded. Laboratory findings including high-sensitive Troponin-T showed normal values. Baseline pro brain natriuretic peptide (proBNP) was slightly elevated with 589 ng/l (Ref. <125 ng/l). Her medication was Metoprolol 47.5 mg controlled release formulation once daily. She was awaiting her elective mitral valve replacement.

Now on admission to our ward, the patient complained of cough, sore throat, and myalgia and had a subfebrile temperature of 38.1°C. Her heart rate was 70 beats per minute, the blood pressure 112/70 mmHg, the respiratory rate 18 breaths per minute and oxygen saturation 99%. A naso-pharyngeal swab for reverse-transcriptase polymerase chain reaction testing to detect severe acute respiratory syndrome coronavirus 2 reported positive the same day. No antibody testings against severe acute respiratory syndrome coronavirus 2 was done and urine and faeces were not tested. Droplet and contact precautions were initiated, and she was referred to our infectious disease department for isolation and monitoring. Two days later, the temperature had normalised. Her heart rate, breathing rate, oxygen saturation and blood pressure remained unchanged over the course of 8 days on the ward. Continuous heart rhythm monitoring showed no changes from baseline arrhythmia. Total white cell count decreased to 4 × 10^9^/l, while lymphocytes increased to 48.1% on day 6. C-reactive protein remained low. The proBNP value increased to 1250 ng/l on day 2, as a sign of cardiac distress, and decreased to 270 ng/l on day 4. There were no signs of myocardial involvement as high-sensitive Troponin-T values remained normal, and no changes on electrocardiogram were seen. Transthoracic echocardiography showed unchanged severity of mitral valve pathology and no worsening of myocardial function. Since discharge on day 10, the patient remained stable and showed no worsening of cardiac or pulmonary findings. Naso-pharyngeal swabs for severe acute respiratory syndrome coronavirus 2 were still positive at discharge. Until the most recent follow-up 4 weeks after discharge, the patient showed no clinical signs of “Multisystem Inflammatory Syndrome in Children” (MIS-C).

Children infected with severe acute respiratory syndrome coronavirus 2 mostly present with a mild phenotype of disease.^1^ In adults, cardiac co-morbidities are known risk factors for a severe course of coronavirus disease 2019 infections. So far, only little data are available concerning the risk profile of children and teenagers with predisposing cardiac conditions like CHDs.^2^ Available data suggest that children with cardiac and other co-morbidities also have a higher chance of severe coronavirus disease 2019^3^ but almost no mortality has been reported. This case illustrates that even with severe cardiac disease with mitral valvular stenosis after valve replacement, pulmonary hypertension and arrhythmia children can have an oligosymptomatic coronavirus disease 2019 infection without cardiac or pulmonary deterioration. Nevertheless, timing of the planned valve replacement had to be postponed by several weeks, because of persistent viral load and fear of complications. The intervention has not been performed yet, so no comment about perioperative complications can be given. More data concerning this highly vulnerable group of patients are needed to improve official guidelines.
